# Recycling endosomes associate with Golgi stacks in sea urchin embryos

**DOI:** 10.1080/19420889.2020.1761069

**Published:** 2020-04-30

**Authors:** Syara Fujii, Tatsuya Tago, Naoaki Sakamoto, Takashi Yamamoto, Takunori Satoh, Akiko K. Satoh

**Affiliations:** a Program of Life and Environmental Science, Graduate School of Integral Science for Life, Hiroshima University, Hiroshima, Japan; b Program of Mathematical and Life Science, Graduate School of Integral Science for Life, Hiroshima University, Hiroshima, Japan

**Keywords:** Recycling endosome, trans-Golgi network, sea urchin, Golgi stack, embryo, mRNA injection

## Abstract

The trans-Golgi network (TGN) and recycling endosome (RE) have been recognized as sorting centers, the former for newly synthesized and the latter for endocytosed proteins. However, recent findings have revealed that TGN also receives endocytosed materials and RE accepts newly synthesized proteins destined to the plasma membrane. Recently, we reported that in both *Drosophila* and microtubule-disrupted HeLa cells, REs are associated with the trans-side of Golgi stacks. REs are highly dynamic: their separation from and association with Golgi stacks are often observed. Importantly, a newly synthesized cargo, glycosylphosphatidylinositol-anchored-GFP was found to be concentrated in Golgi-associated REs (GA-REs), while another cargo VSVG-GFP was excluded from GA-REs before post-Golgi trafficking to the plasma membrane. This suggested that the sorting of cargos takes place at the interface of Golgi stacks and GA-REs. In this study, we demonstrated that REs could associate with Golgi stacks in sea urchin embryos, further indicating that the association of REs with Golgi stacks is a well-conserved phenomenon in the animal kingdom.

## Introduction

Golgi stacks are composed of several flattened membrane compartments called cisternae, which are highly polarized: newly synthesized proteins integrated to the endoplasmic reticulum enter from cis cisternae, which gradually matures to trans-cisternae. In the classical view, trans-Golgi networks (TGNs) residing at the trans-ends of the Golgi stacks act as a sorting center for newly synthesized proteins [1,2], whereas the recycling endosomes (REs) gathering near the nucleus recycle endocytosed materials back to the plasma membrane [3,4]. However, recent reports suggest that both TGNs and REs are considered as the hubs of endocytic and exocytic pathways, involved in the sorting of the endocytic and exocytic proteins [5,6].

Recently, we reported that, in both *Drosophila* and microtubule-disrupted HeLa cells, REs can exist in two distinct states: Golgi-associated REs (GA-RE) and Golgi-independent REs (free-REs) [7]. Upon assessing the association between Golgi stacks and REs, we revealed that more than 70% of Golgi stacks are accompanied by REs; however, a substantial amount of free-REs were also found to exist. Using the super-resolution confocal live imaging microscopy (SCLIM) [8,9], we examined the dynamic relationship between GA-REs and free-REs, and observed that REs can occasionally separate and thereafter re-associate with the Golgi stacks. Furthermore, REs can themselves separate from or associate with each other. Thus, we could state that REs are highly dynamic, and GA-REs and free-REs are inter-changeable. We also demonstrated that the newly synthesized GPI-anchored cargo temporarily localizes to GA-REs before reaching the plasma membrane; however, newly synthesized vesicular stomatitis virus (VSV) G protein molecules (VSV-G) are excluded from GA-REs, and seem to be transported to the plasma membrane directly from the Golgi stacks. These results further suggested that GPI and VSV-G might be sorted at the interface between the trans-side of Golgi stacks and GA-REs. In this study, we determined the generality of RE-association with Golgi stacks using the sea urchin embryos.

## Materials and methods

### Animals and embryos

Adults of the Japanese sea urchin, *Hemicentrotus pulcherrimus*, were collected from the Seto Inland Sea or Tateyama Bay. Eggs and sperm were obtained by coelomic injection of 0.55 M KCl. Fertilized eggs were subsequently cultured in filtered seawater at 11°C. About 16 h post fertilization, nocodazole was added to the seawater containing fertilized eggs at a final concentration of 20 nM.

### Construction and injection of mRNA

For *in vitro* transcription of mRNA, the DNA templates were amplified from the plasmids, pMT-GalT-EGFP-T2A-tdTomato-Rab11 and pMT-GalT-EGFP-T2A-tdTomato-Vamp3, by using the KOD-Plus-Neo DNA polymerase (Toyobo, Japan) and the following primers: T7-MT-F (5ʹ-TAATACGACTCACTATAGGGtcagcagcaaaatcaagtgaatcat-3ʹ) and SV40-pA (5ʹ-ttttttttttttttttttttttttttttttcactgcattctagttgtggtttgt-3ʹ). Using 1 µg of DNA templates, capped and poly-A tailed mRNAs were transcribed by using the HiScribe T7 ARCA mRNA Kit (with tailing; NEB), and then the resultant mRNAs were purified by using the Zymo RNA Clean & Concentrator-25 (Zymo Research) and eluted in water. The mRNA was mixed with glycerol at a final concentration of 40%, and then was used for microinjection at a final concentration of 5 ng/μl as described previously [10].

### Image acquisition and analysis

Sea urchin embryos were observed under the FV3000 confocal microscope equipped with UPLSAPO60XS2 silicone immersion objective at 60 × magnification. To minimize bleed-through of fluorescence emission for each sample, signal for each of the three fluorescent proteins was captured sequentially. Images were processed following the Guidelines for Proper Digital Image Handling using Fiji, Affinity photo, and/or Adobe Photoshop CS3 (Adobe, San Jose, CA, USA).

## Results and discussion

In our recent findings, we unexpectedly found that REs associate with Golgi stack, but so far we observed this phenomenon only in *Drosophila* and human cultured cells. Therefore, to understand whether this association between REs and Golgi is well conserved in the animal kingdom, it was important to investigate whether this phenomenon also occurs in evolutionarily distant organisms from both, *Drosophila* and human. However, it is challenging to perform the indirect-immunofluorescent experiments using the Golgi stack and RE markers in the evolutionarily distant organisms. This is mainly because the currently available antibodies against the Golgi stack and RE resident proteins of human or *Drosophila* would fail to cross-react with the orthologs in other organisms. Instead, another strategy involving the microinjection of mRNA into the fertilized eggs or early embryos would be a more promising method to visualize the Golgi stacks and REs, although the availability of fertilized eggs and the optimized microinjection-methods are limited. Sea urchin, an invertebrate belonging to the deuterostome lineage, is considered as an excellent model system to visualize the Golgi stacks and REs because the method of RNA-injection into the fertilized eggs has previously been well established.

**Figure 1.**
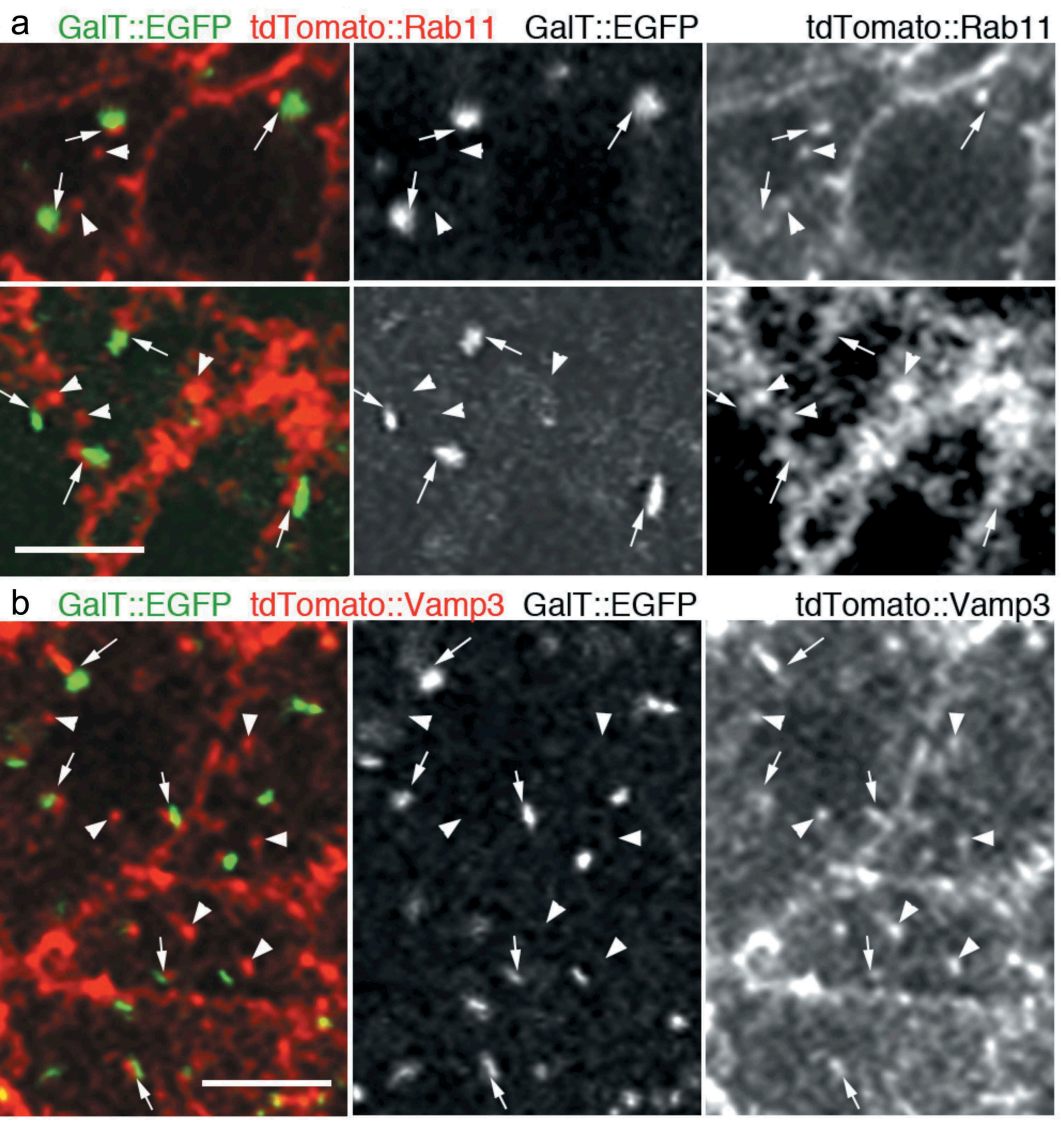
REs associate with the trans-side of Golgi stacks in sea urchin embryos

In this study, we injected mRNAs coding the trans-Golgi marker GalT::EGFP, and the most widely-accepted RE marker tdTomato::Rab11 [11–13] into the fertilized sea urchin eggs and then incubated them for 16 h at 11°C, when embryos were supposedly in the early blastula stage. It has been reported that the scattered Golgi stacks turn into aggregates during the early blastula stage [14]. As it would be difficult to assess if each of the Golgi stack is associated with RE in this aggregated form, we further incubated the mRNA-injected sea urchin embryos with 20 nM nocodazole for more than 2 h to maintain the separation of Golgi stacks as previously reported [14]. In this condition, numerous Golgi stacks and foci of REs were found to be scattered in the cytoplasm. We observed that most of the GalT::EGFP signals were accompanied by the foci labeled with tdTomato::Rab11 (Figure 1(a); Arrows). Conversely, there were foci of tdTomato::Rab11 that were found without the adjacent Golgi marker GalT::EGFP (Figure 1(a); Arrowheads). We also assessed the association of Golgi stacks and REs using another well-established RE marker tdTomato::Vamp3 by using the mRNA-injected and nocodazole-treated embryos [15]. Similarly, most of the GalT::EGFP were found to be associated with foci labeled with tdTomato::Vamp3 (Figure 1(b); Arrows), but we also observed Golgi-independent foci of tdTomato::Vamp3 (Figure 1(b); Arrowheads). These results indicate that in sea urchin embryos, there are two-kinds of REs: GA-REs and free-REs.

(a) Sea urchin embryo expressing GalT::EGFP (green) and tdTomato::Rab11 (red) or (b) GalT::EGFP (green) and tdTomato::Vamp3 (red). Arrows are indicating the GA-REs, and arrowheads are indicating the free-REs.Scale bars: 5 μm (for both A and B).

Here, we demonstrated that REs associate with the trans-side of the Golgi stack in an invertebrate belonging to the deuterostome lineage, the sea urchin, which is an evolutionarily distant organism from *Drosophila* and humans. This further indicates that the association of REs with Golgi stacks is a well-conserved phenomenon in the animal kingdom.
